# Mechanical Properties of Ultra-High-Performance Concrete with Steel and PVA Fibers

**DOI:** 10.3390/ma17235990

**Published:** 2024-12-06

**Authors:** Ana Elisabete P. G. A. Jacintho, André M. dos Santos, Gilvan B. Santos Junior, Pablo A. Krahl, Grazielle G. Barbante, Lia L. Pimentel, Nádia C. S. Forti

**Affiliations:** 1 Urban Infrastructure System Program, Pontifical Catholic University of Campinas (PUC-Campinas), Campinas 13086-099, Brazil; andre-mendes2007@hotmail.com (A.M.d.S.); graziellegomesbarbante@gmail.com (G.G.B.); lialp@puc-campinas.edu.br (L.L.P.); nadia.cazarim@puc-campinas.edu.br (N.C.S.F.); 2 Semi Árido Federal University, Mossoró 59625-900, Brazil; gilvan.bezerra@ufersa.edu.br; 3 Department of Civil Engineering, Mackenzie Presbyterian University, Av. Brasil, 1220-Jardim Guanabara, Campinas 13073-148, Brazil; pabloaugustokrahl@gmail.com

**Keywords:** UHPC, fibers, compression strength, tensile strength, elasticity modulus, CCRD

## Abstract

Ultra-high-performance concrete (UHPC) has gained worldwide popularity due to its high mechanical performance. This research studied the influence of adding a mixture of two fibers (steel and PVA) on the compressive strength, modulus of elasticity, and flexural tensile strength of UHPC. The mixtures were prepared by adding steel fibers and PVA fibers using a standard procedure defined in the research, which is the time to mix the dry materials and the time to mix the admixture and water. The Central Composite Rotational Design (CCRD) methodology was used for the experimental design of the compressive strength and longitudinal deformation modulus tests. The results were analyzed using statistical software to investigate the influence of fibers on these two mechanical properties of UHPC. With this technique, an optimized design for the study of flexural tensile strength was arrived at. It was found that the standardized equations for the modulus of elasticity, directed to conventional concrete and high-strength concrete, are inadequate for estimating the modulus of UHPC in this research. Statistical analysis indicated that the range of fiber amounts analyzed did not significantly affect the compressive strength and modulus of elasticity. Regarding the optimized mixture, its flexural tensile strength indicated that the fiber content should be higher for UHPC to be suitable for structural use.

## 1. Introduction

Ultra-high-performance concrete (UHPC) is a unique type of concrete that has been studied worldwide for the last four decades. It stands out due to its particular characteristics, such as a compressive strength of over 120 MPa, the absence of coarse aggregate in some cases, a high amount of fine materials, and plasticizing additives that improve the rheology of the cement matrix. UHPC with fiber additions, also known as ultra-high-performance fiber-reinforced concrete (UHPFRC), has notable characteristics such as increased tensile strength and post-cracking ductile behavior.

The global recognition of UHPC is evident from the fact that several international standards have already been published and enforced for its production and structural use. These standards not only deal with the material’s properties but also provide design procedures. There are many types of fibers, and it is necessary to conduct more research to know what kinds of fiber work better in the UHPC, or if it is possible to addict more than one fiber and to obtain good behavior in its hardening tensile strength. There are experimental studies on hybrid UHPC, but codes still do not address this topic, emphasizing the importance of the present study [1,2,3,4,5,6]. From the literature review, the hybrid combination was from trial and error, and no optimization analysis was performed to determine the best combination of different fibers in UHPC, which is a goal of the present study.

Table 1 provides an overview of some of these standards and the countries where they have been published, further highlighting UHPC’s global reach.

Ultra-high-performance concrete (UHPC) in its present form became commercially available in the United States in about 2000. The Federal Highway Administration (FHWA) began investigating the use of UHPC for highway infrastructure in 2001 and has been working with state transportation departments to deploy the technology since 2002 [12]. Because of the improvement in the use of this material worldwide, much research has been conducted since then about the mix proportions [18,19,20,21,22,23,24,25] and structural behaviors [26,27,28,29,30,31].

This research aims to characterize UHPC based on its compressive strength and modulus of elasticity using two types of fibers: steel and PVA. This characterization used a statistical program based on Central Composite Rotational Design (CCRD) to find an optimized mix. So the tensile strength of this optimized mix was analyzed for its hardening behavior.

## 2. Materials and Experimental Program

**Figure 1:**
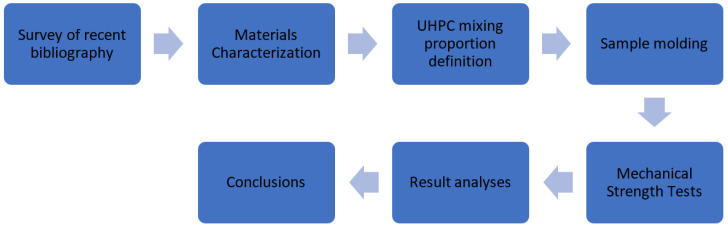
Methodology Scheme.

This study aims to characterize UHPC based on its compressive strength, modulus of elasticity, and tensile strength. Figure 1 depicts the research steps.

During the initial stages of the experiment, a survey of the existing literature was conducted, followed by mechanical tests to determine the compressive strength and modulus of elasticity of the materials under investigation. The Central Composite Rotational Design (CCRD) was then used to plan and conduct compression tests to determine the optimized composition for investigating UHPC tensile strength with steel and PVA fibers.

This optimized composition was then used to mold cylindrical and prismatic specimens for bending tensile strength tests while monitoring the materials’ compressive strength and modulus of elasticity. Table 2 summarizes the sizes and quantities of the specimens used and the tests carried out, including their respective ages.

### 2.1. Materials Characterization

The production of UHPC requires a more stringent selection of materials to achieve the desired physical and mechanical characteristics [31]. This research used fine quartz sand (with a maximum diameter of 0.516 mm) and quartz powder (with a maximum diameter of 0.052 mm). Silica fume in powder format was used as a mineral addition. Polycarboxylate ether additives (superplasticizers) were used to keep the water-to-cement (*w*/*c*) ratio low. The cement used was CP V-ARI type (Type III in ASTM C 150/150M-21 [32]).

**Figure 2:**
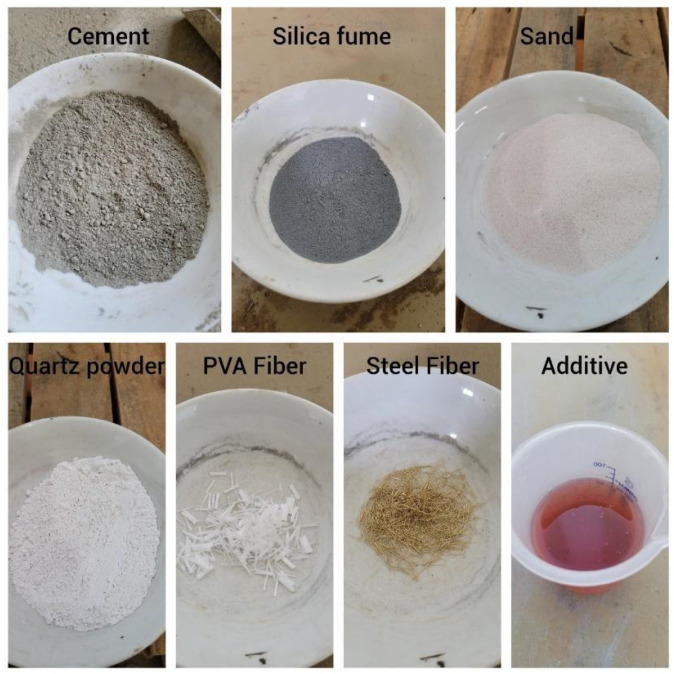
UHPC component materials.

The steel fibers used were 13 mm long and 0.2 mm in diameter, with a specific mass of 7.85 g/m^3^, tensile strength of 2160 MPa, and elastic modulus of 210 GPa. Similarly, the PVA fibers used in this research were 12 mm long and 0.04 mm in diameter, with a specific mass of 1.30 g/m^3^, tensile strength of 1600 MPa, and elastic modulus of 41 GPa. The materials of mixing proportion are presented in Figure 2.

### 2.2. Mixing Proportion

The proportion used in this research was adapted from [33], which used the material composition from Vigneshwari, Arunhachalam, and Angayarkanni (2018) [34] as a base reference in the proportion of 1: 1.1: 0.1: 0.194: 0.19: 0.04 (cement: sand: quartz powder: silica: *w*/*c*: additive).

The material studied was an ultra-high strength concrete reinforced with a blend of steel fibers and PVA. However, using PVA fiber can reduce the resistance of UHPC, as explained in [31]. Thus, the amount of PVA fiber used in this research was considered small. The PVA fiber content varied from 0% to 0.38%, and the steel fiber content varied from 1% to 2%. This variation was defined using the CCRD with factorial $2^{2}$ to analyze the influence of the variation in addition to fiber contents on the compressive strength and tensile strength in bending.

Equation (1) indicates the amount of sample required for an adequate analysis with developments of tools such as surface and results curve.

(1) $$n=2^{k}+2\cdot k+p$$

where:

*n* = sample size;

*k* = amount of independent variables;

*p* = amount of central points.

The analysis utilized three central points and two independent variables: the steel and PVA fiber percentage inside composition. The minimum sample size required for analyzing the response surface in the Statistica 10 software was *n* = 11.

In Table 3, it is possible to verify the distribution of these coded variables.

### 2.3. Mixing and Molding Process

The first mix was created by following Sumitomo’s study (2022) [33], where he adapted the process used by Hiremath and Yaragal (2017) [35]. This process was named Mixing Process 1.

In the production, the amount of water and additives were divided into four equal parts, which were then incorporated into the fine materials. These fine materials were divided into different parts. Mixing Process 1 involved the following steps with each mixing time:

1. 50% cement + 100% silica fume + 50% water (5 min).
2. 25% cement + 50% sand +50% additive +25% water (5 min).
3. 25% cement (5 min).
4. 50% sand + 100% quartz powder + 50% additive + 25% water (5 min).
5. 100% fibers (5 min).

**Figure 3:**
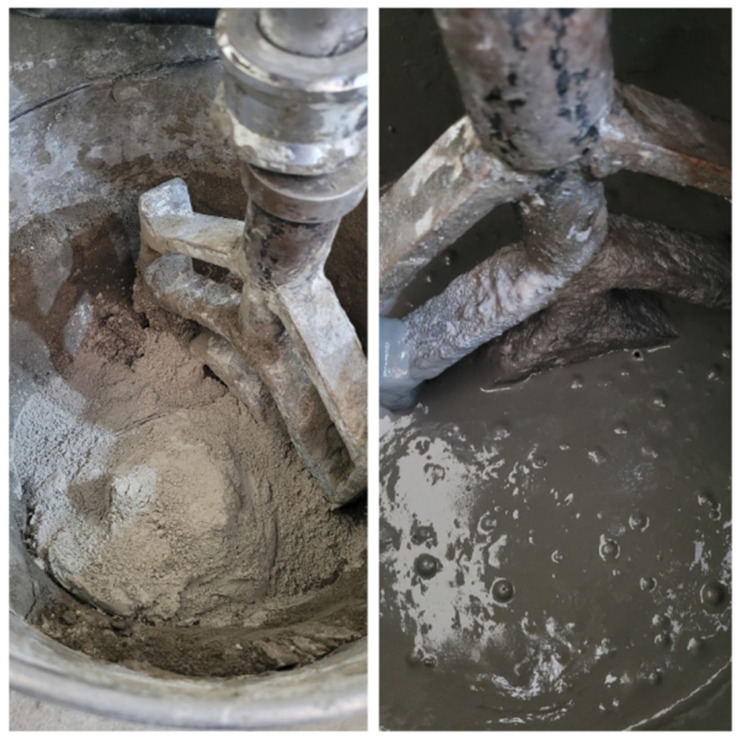
UHPC Mixing Process 1.

Manual compaction was carried out after mixing and molding the 7.5 cm × 15 cm specimens. Figure 3 shows the steps involved in producing UHPC using the mixer.

A new process adaptation was introduced, significantly improving the production of the last mixes and achieving a better rheology. This process was called Mixing Process 2. The fine materials were combined in a bucket using a beater attached to a drill. The mixture was then transferred to a three-stage mixer. Mixing Process 2 involved the following steps with each mixing time:

1. 1/3 of fine materials (sand, cement, quartz powder, and silica fume) + 50% water + 50% additive (5 min);
2. 1/3 of fine materials (sand, cement, quartz powder, and silica fume) + 25% water + 25% additive (5 min);
3. 1/3 of fine materials (sand, cement, quartz powder, and silica fume) + 25% water + 25% additive (5 min);
4. 100% fibers (3 min);
5. use of a drill with a beater (2 min).

The entire process, from the initial mixing to the final molding of the cylindrical specimens, took approximately 50 min. Mixing Process 2 consisted of 20 min of mixing time in the mixer and the final stage with the drill, while Mixing Process 1 took a little over half an hour. It resulted in a longer UHPC flowable time, while molding the specimens reduced mixing time. However, no change in concrete strength was observed due to the change in the mixing procedure. This process was carried out in a small mixer, whereas in industry, this process is carried out in large mixers, whose preparation time can be drastically reduced.

The difference between the two processes was in the total time to prepare the mixture, in which Process 2 took less time than Process 1. Regarding the results of mechanical properties, there was no difference noted due to the mixing processes.

This material is used in industrialized construction, for example, in precast structural elements, mainly for bridges, which is why the mixture made in large volumes is prepared in large mixers with high energy capacity. Also, scale-up fiber-reinforced materials, like UHPC, must comply with standardized rheology requests (self-consolidation) and guarantee fiber dispersion in such mixers for adequate casting and compaction and does not change the hardened properties.

**Figure 4:**
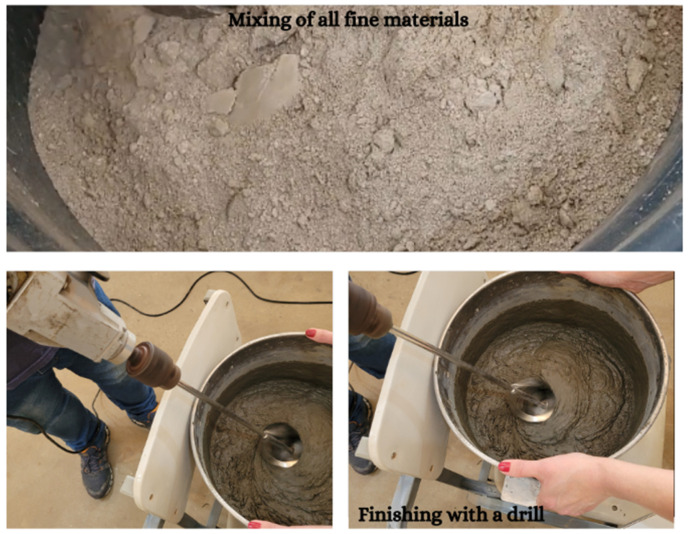
UHPC Mixing Process 2.

Figure 4 depicts mixing fine materials in the bucket and finishing with the drill and mixer.

Due to the large volume of concrete, three castings were used to mold the prismatic specimens, following the second procedure described previously.

**Figure 5:**
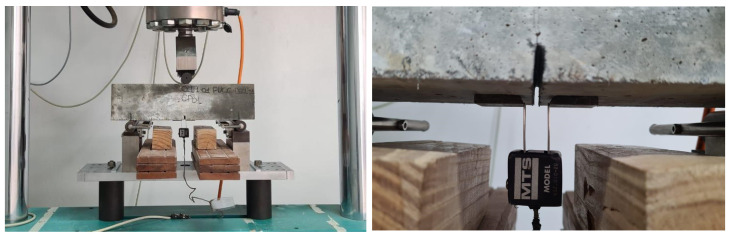
The 3-point test.

**Figure 6:**
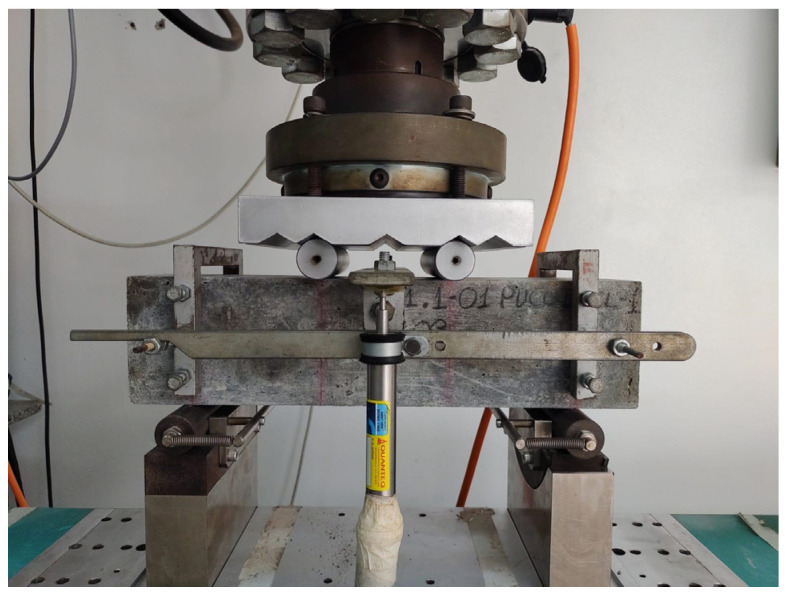
The 4-point bending test.

Six prismatic specimens with dimensions of 10 cm × 10 cm × 40 cm were used for the 3- and 4-point bending tensile strength tests (see Figure 5 and Figure 6). Three specimens were used in the 4-point bending test, and the remaining three were used in the 3-point test. The tests were conducted with deformation control, following the Recommended Practice of Jacintho et al. (2022) [16] and the French standard NF P18 470 (2016) [9].

## 3. Results and Discussions

### 3.1. Compression Strength Tests Results

Table 4 presents the compressive strength results for the 11 mixes at 7 and 28 days, as well as the elasticity modulus at 28 days and the fiber content. On average, the resistance of UHPC at 7 days is 72% of that at 28 days.

### 3.2. Elasticity Modulus

Table 4 presents the experimental results of the elasticity modulus at 28 days as the compressive strength.

Experimental results were compared to the estimated values provided by national and international standards for conventional and high-strength concrete. Additionally, the recommendations presented by Graybeal (2019) [36] and Sritharan (2003) [37] were compared, as these are specific to UHPC. The applied standards include NBR 6118:2023 [38] (Class I and Class II), ACI 318 [39], FIB Model Code [40], and Eurocode 2 [41]. The equations used to estimate the modules are presented in Table 5.

To estimate the elasticity modulus values according to scientific articles and standards, the characteristic compressive strengths ($f_{ck}$ as per NBR 6118:2023 [38] and $f_{c}^{\prime }$ as per ACI 318 [39]) were initially calculated, as shown in Table 6.

The resulting theoretical values are presented in Table 7.

### 3.3. CCRD Analysis

The experimental data were entered into the Statistica 10 software for a more in-depth analysis of which factors influenced the compressive strength and elastic modulus. The response evaluated the use of two types of fibers and whether the results had an acceptable dispersion. Constructing the response surfaces of the CCRD was possible.

**Figure 7:**
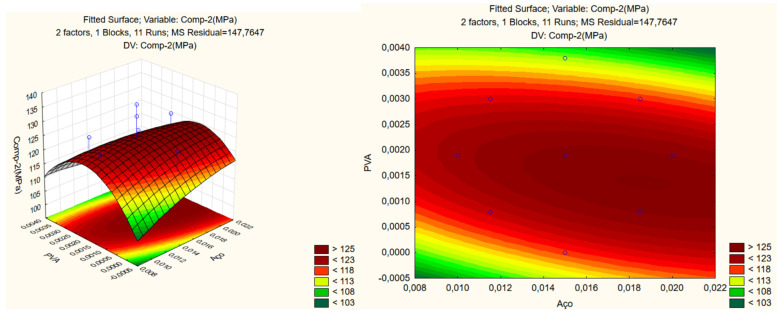
Response surface of compression strength

**Figure 8:**
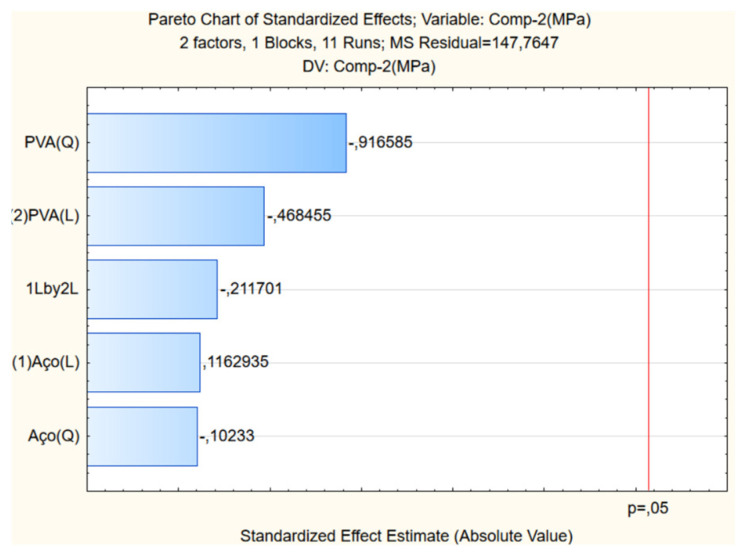
Pareto for compression strength.

Figure 7 presents the CCRD response for compressive strength and the influence of variables on the surface’s construction, which is given through Pareto analysis (Figure 8).

**Figure 9:**
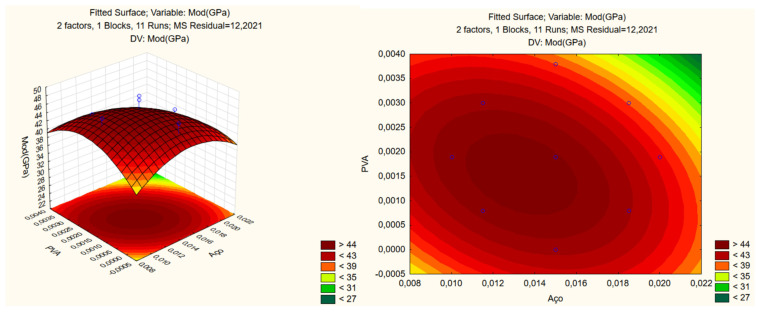
Response surface of elasticity modulus.

**Figure 10:**
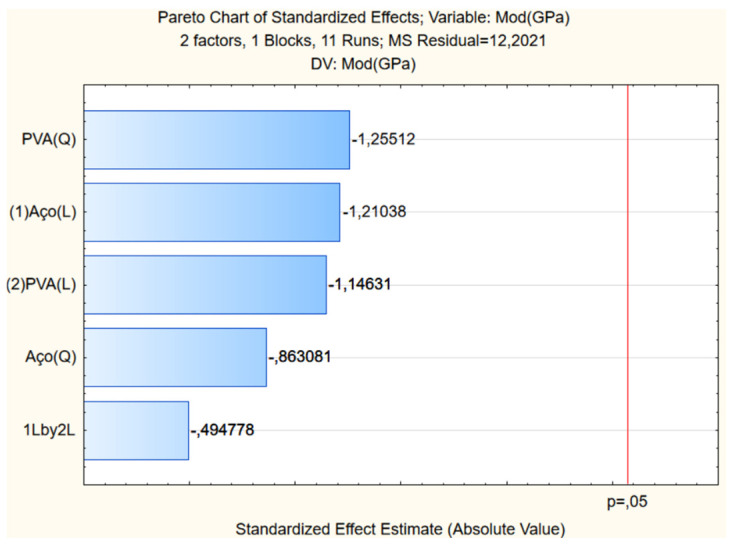
Pareto of elasticity modulus.

As compressive strength, Figure 9 depicts the impact of steel and PVA fibers on the elastic modulus of UHPC. The Pareto analysis highlights the influence of variables on surface construction (Figure 10).

Table 8 presents the ANOVA results for the compressive strength and modulus of elasticity of eleven UHPC mixes, as analyzed by Statistica 10 software.

The Pareto chart and the ANOVA analysis concluded that the variation in steel and PVA fibers used in this research did not significantly influence the compressive strength and elasticity modulus.

This may have happened due to the different content of PVA fibers compared to the content of steel fibers added to the cement matrix. The content of steel fibers added was much higher than the content of PVA fibers. The study of UHPC with increasing PVA fiber content while keeping the steel fiber content constant will be the continuation of this research.

### 3.4. Elasticity Modulus Prediction Analysis

Equation (2) was utilized to compare the estimated values of elasticity modulus obtained from approximations with the experimental average values. The results are presented in Table 9, which shows the proximity or distance of these estimates by standards and articles from the experimental results. This methodology was conducted similarly to the study by Jacintho et al. (2020) [42], providing a better understanding of the relationship between the estimates and experimental values.

As expected, the Class I concrete equation NBR 6118:2023 [38] resulted in an average discrepancy of about 28% from experimental values. For Class II concretes (with high compressive strength), the elasticity modulus values obtained through experiments were, on average, 14% lower than those estimated using the NBR 6118:2023 equation [38]. This difference is lower than the values estimated with the equation for Class I concrete.

The experimental elasticity modulus values were approximately 11% lower than those estimated with the equations of ACI 318 (2014) [39], Model Code (2010) [40], and Eurocode 2 (2010) [41] (for high-strength concrete), which is slightly lower than NBR 6118:2023 [38] Class II.

The equations proposed by Graybeal (2019) [36] and Sritharan (2003) [37] for estimating the elasticity modulus of UHPCs provided results that were closest to the experimental values and also in favor of safety. These results highlight the unique behavior of this material and point out that parameters must be obtained through experimental results or specific approximations.

### 3.5. Tensile Strength

In this research, the tensile strength of UHPC was studied by defining a mix with the appropriate amount of optimized steel and PVA fibers to perform flexural tests to study the tensile strength. It was necessary to make a concrete volume bigger than that used in the compressive strength tests. So, experimental planning in compressive tests was used to find an optimized steel and PVA fibers content mix.

The quantity was determined using Statistica 10 software, which was used to construct the response surface for compressive strength. The fiber contents have been optimized to 1.86% steel and 0.14% PVA fibers. With this mixture, the expected compressive strength is 125.0813 MPa. At 28 days, the experimental compressive strength was 132.01 MPa, and the elasticity modulus was 45.22 GPa.

**Figure 11:**
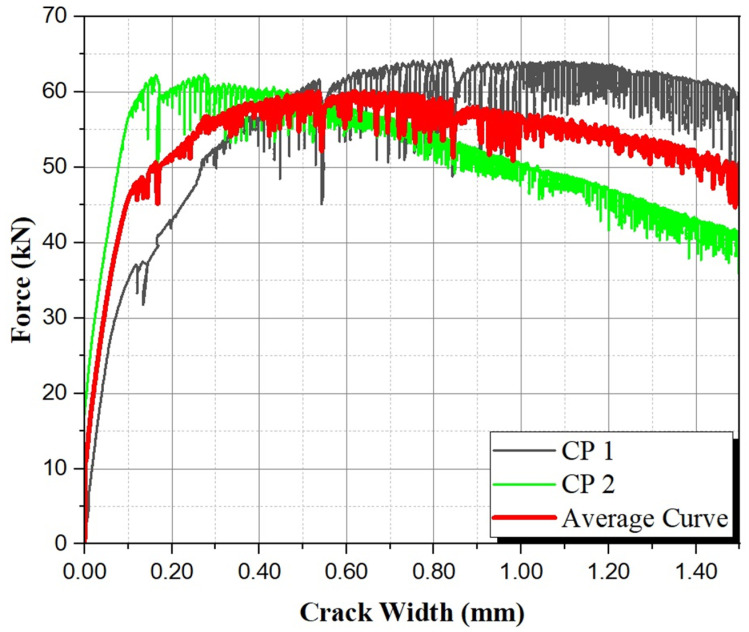
Experimental curves obtained in the 4-point bending tests.

According to Jacintho et al. (2022) [16], to determine the tensile strength in the elastic phase, the mean curve generated by the Origin Lab 2023 software was utilized from the 4-point bending test curves, as shown in Figure 11.

UHPC has a dense matrix that makes it a high-performance material due to its high resistance to aggressive environments (Li et al., 2020) [43] and improved interaction with fibers controlling better crack opening, resulting in the hardening behavior observed in Figure 11, even with straight fibers. Its pore size distribution is well known to be on the scale of nanometers, with no capillarity pores (Schmidt and Fehling, 2005) [44]. Because of this, codes recommend smaller reinforcement covers for UHPC structural elements (NF P18 710 [10]).

Two specimens were used for the analysis. Equations (9) and (10) were used to determine flexural and elastic tensile strength, respectively. Table 10 presents the equations’ parameters and the results.

(9) $$f_{ct,fl}=\frac{3.Fl}{b.a}\;(MPa)$$

(10) $$f_{ct,el}=f_{ct,fl}\left[\frac{K.a^{0,7}}{1+K.a^{0,7}}\right](MPa)$$

with k = 0.08 (NF P18 470, 2016) [9].

The yield point was obtained from the graph generated using the average response of two prismatic specimens in a 4-point bending test.

**Figure 12:**
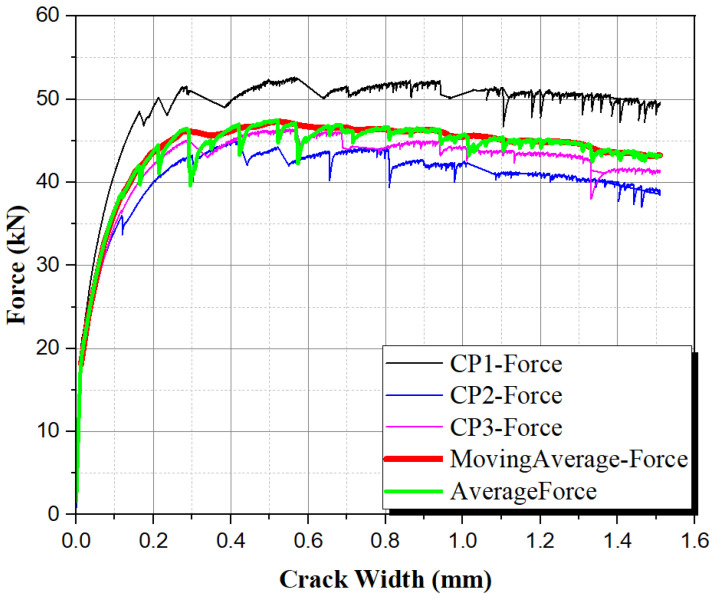
Experimental curves obtained in the 3-point bending tests with the average curve and the moving average curve.

Figure 12 presents the curves generated in the 3-point bending tensile strength test, with the average curve generated in the Origin Lab 2023 software. The “x” axis deformation is the crack opening measured during the test. Also included in the graph is the curve that was created using the moving average of the data. It is based on the recommendations of the French standard NF P18 470 (2016) [9] and Jacintho et al. (2022) [16]. The moving average was calculated in Excel, and the graph was created using the Origin Lab software.

Strain hardening behavior is observed in 3-point and 4-point bending tests. The hybrid solution with fibers with different diameters controls cracking at two scales, promoting a synergistic effect (Banthia and Gupta 2004) [45], i.e., an improved behavior compared to the composite with one fiber type. Therefore, due to the high loading variability in real applications, crack opening at different service load levels will be better controlled with hybrid UHPC. This would increase the durability and life span of structures made with such material, reducing repair, maintenance, and retrofit costs. Also, the rational application of UHPC is currently being investigated, for example, in the punching failure surface on flat slabs (de Souza et al. 2021) [25], increasing the substantially of the shear behavior and optimizing the structural elements to reduce the consumption of natural resources and cement (Feghali et al. 2024) [46]. Therefore, both approaches can reduce environmental impacts and costs to balance the whole life cycle of structures.

### 3.6. Inverse Analyses by NF P18 470 (2016) [9]

**Figure 13:**
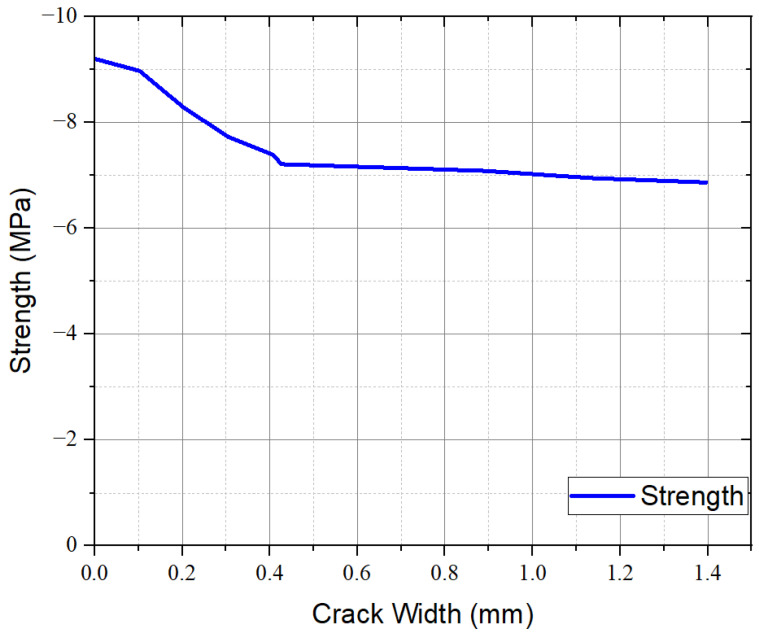
Inverse analysis of the 3-point bending test.

The results of three-point bending were used to determine the UHPC tensile response according to the French code. It was conducted using the Maple 2023 software, following the equations prescribed in the French standard NF P18 470 (2016) [9]. The formulation presented in the code is based on the equations of Casanova (1995) [47], which corresponds to a multilinear inverse analysis that considers the region in which cracking occurs in the prism as a plastic hinge whose curvature is represented by a parabola and the remaining regions a constant curvature. Also, the crack opening presents a linear profile. Casanova (1995) [47] considers that plastic hinges have two times the crack depth, which varies during the analysis. Finally, the concrete above the crack is considered to have linear behavior. Therefore, stress integration can be performed in the section. The result is presented in Figure 13, which was created using the Origin Lab 2023 software.

The peak tensile strength point (f_ctf_), obtained by inverse analysis, was 6.27 MPa. According to NF P18 710 [10], if the ratio of f_ctf_ divided by 1.25 is lower than f_ct,el_, the material is classified as T1, which indicates a softening behavior. For this optimized concrete, the f_ctf_ divided by 1.25 is 5.02 MPa, which is lower than 9.32 MPa, and thus, it is classified as T1 and exhibits a softening behavior. It occurred despite adding 1.86% steel fibers and 0.14% PVA fibers.

### 3.7. Discussion of Results

It was made of 11 UHPC mixtures, where the proportion of dry materials remained constant and the content of steel fibers and PVA were varied. The average compressive strength for each mix ranged from 106 MPa to 135.86 MPa, with the variation going from 0.69 MPa to 6.98 MPa. The modulus of elasticity obtained for each of the 11 mixes had values that ranged from 38.23 GPa to 48.24 GPa, with the variation going from 0.64 GPa to 5.15 GPa.

CCRD analysis was applied to find a mix with the optimized amount of steel fibers and PVA. For this optimized mix, the average compressive strength of UHPC was found to be 132.01 MPa, and the modulus of elasticity of 45.22 GPa. For this optimized design, 3-point and 4-point prism bending tests were performed, and by applying the Inverse Analysis proposed by NF P18 470 [9], it was possible to find the tensile strength at the elasticity limit with the value of 9.32 MPa and the post-cracking strength with the value of 6.27 MPa. With the tensile strength results, it can be concluded that this is a UHPC classified as T1* by the French standard NF P18 710 [10], with post-cracking softening behavior. The values of the modulus of elasticity obtained experimentally were compared with values estimated by the Brazilian standard and by international standards, as well as with equations proposed by researchers to estimate the longitudinal deformation modulus specifically for UHPC, but which are not standardized. The equations for estimating the modulus proposed by Graybeal (2019) [36] and Srithaman (2003) [37] presented values closer to those obtained experimentally.

This study was limited to the use of two types of fibers in UHPC, steel and PVA. Future studies could use more than two types of fibers and a greater variation in the amount of fibers added to the matrix.

## 4. Conclusions

The study investigated the mechanical behavior of UHPC with steel and PVA fibers. The researchers added varying amounts of steel fibers, between 1% and 2%, and PVA fibers ranging from 0% to 0.38% while keeping the base mix of UHPC constant.

The highest average compressive strength values were observed in mix T7, which had a steel fiber content of 1.50% and PVA fibers of 0.19%. In this mix, the highest value in an individual specimen was 146.33 MPa, with an average compressive strength of 135.86 MPa.

In addition, the study verified the elastic modulus of the different UHPC mixes. Mix T6 had the highest value, 48.24 GPa. In conjunction with the mixes T5 and T7, which had the same amount of fibers at 1.5% steel and 0.19% PVA, these were the three central points of the CCRD.

The researchers conducted a CCRD analysis using the Statistica 10 software. They found that varying the quantity of fibers (PVA and steel) did not significantly affect the compressive strength and elastic modulus within the range of fiber quantities used in this research.

The elastic modulus values estimated by the standards were compared with the experimental values. The equations of NBR 6118:2023 [38] for Class I and Class II concretes produced values farthest from the experimental values. The estimated values from ACI 318 (2014) [39], Model Code (2010) [40], and Eurocode 2 (2010) [41] were higher than the experimental values by about 20%, being slightly lower than NBR 6118:2023 [38] Class II. The equations proposed by Graybeal (2019) [36] and Sritharan (2003) [37] were more in favor of safety as they produced values closer to the experimental values. Both equations were developed specifically for UHPCs.

The 4-point bending test showed that the elastic tensile strength (f_ct,el_) of 9.32 MPa was within the expected range for UHPC. The minimum strength for UHPC is 7 MPa, according to Jacintho et al. (2022) [16]. On the other hand, the 3-point bending test, using the inverse analysis, showed that the f_ctf_ was lower than the f_ct,el_. It means that the concrete produced is classified as T1 (softening), according to French standards NF P18-470 (2016) [9] and NF P18-710 (2016) [10].

This study was limited to the use of two types of fibers in UHPC, steel and PVA. Future studies could use more than two types of fibers and a more significant variation in the amount of fibers added to the matrix.

## Figures and Tables

**Table 1:** Standards and technical recommendations for UHPC.

Year	Publication	From	Country	Subject
Since 2006	FHWA	Federal Highway Administration	E.U.A	UHPC Material Characterization and Behavior
2008	Recommendations for Design and Construction of High-Performance Fiber Reinforced Cement Composites with Multiple Fine Cracks (HPFRCCs) [7]	JAPAN SOCIETY OF CIVIL ENGINEERING—JSCE	Japan	UHPC Material Characterization and Behavior
2014	Design Guidelines for K-UHPC [8]	Korea Institute of Construction Technology (KICT)	South Korea	UHPC Material Characterization and Behavior
2016	NF P18 470 [9]	Association Française de Normalisation	French	Materials
2016	NF P18 710 [10]	Association Française de Normalisation	French	Structural Project
2016	Ultra-high-performance fiber reinforced concrete (UHPFRC)—Materials, design, and execution [11]	Swiss Society of Engineers and Architects	Swiss	Materials and Structural Project
2017	AASHTO LRFD Bridge Design Specifications [12]	AASHTO	E.U.A	UHPC for Bridge
2017	C1856 Standard Practice for Fabricating and Testing Specimens of Ultra High-Performance Concrete [13]	ASTM	E.U.A	UHPC Material Characterization and Behavior
2018	PRC-239-18: Ultra-High-Performance Concrete: An Emerging Technology Report [14]	American Concrete Institute (ACI)	E.U.A	UHPC Material Characterization and Behavior
2019	CSA A23.1:19/CSA A23.2:19 Concrete materials and methods of concrete construction/Test methods and standard practices for concrete [15]	Canadian Standards Association	Canadá	UHPC Material Characterization and Behavior
2022	Prática Recomendada: Concreto De Ultra Alto Desempenho Reforçado Com Fibras (UHPC) [16]	ABECE/IBRACON—Texto Base para ABNT	Brasil	UHPC Material Characterization and Behavior
2022	Fiber Reinforced Concrete—State of the Art [17]	FIB CEB-FIP Bulletin 105	Brasil	UHPC Material Characterization and Behavior

**Table 2:** Number of specimens and UHPC age for each type of test.

Test	Specimen Type	Sample Quantity	
		7 Days	28 Days
Compression Strength	Cylindrical 7.5 cm × 15 cm	3	4
Elasticity Modulus	Cylindrical 7.5 cm × 15 cm		4
Bending Test—3 points	Prismatic 10 cm × 10 cm × 40 cm		3
Bending Test—4 points	Prismatic 10 cm × 10 cm × 40 cm		3

**Table 3:** Variables coded according to CCRD with factorial 2^2^.

Variables	Codification				
	$-\sqrt{2}$	−1	0	1	$\sqrt{2}$
Steel Fibers (Independent)	1.0%	1.15%	1.5%	1.85%	2.0%
PVA fibers (Independent)	0.00%	0.08%	0.19%	0.30%	0.38%
Compression Strength (dependent)	-	-	-	-	-
Elasticity Modulus (dependent)	-	-	-	-	-

**Table 4:** Content fiber, compression strength at 7 days and 28 days, and elasticity modulus at 28 days.

Series	Fibers Content		7 Days Compression Strength		28 Days Compression Strength		28 Days Elasticity Modulus	
	Steel (%)	PVA (%)	Average Strength (MPa)	Standard Deviation (MPa)	Average Strength (MPa)	Standard Deviation (MPa)	Average Modulus (GPa)	Standard Deviation (GPa)
T1	1.00	0.19	60.94	7.37	122.94	2.50	46.14	3.04
T2	1.15	0.08	89.36	2.43	110.13	3.33	41.44	0.71
T3	1.15	0.38	82.89	4.70	124.21	5.57	43.63	1.16
T4	1.50	0.00	91.83	6.71	125.54	3.47	46.08	1.62
T5	1.50	0.19	87.15	3.88	106.00	0.69	40.90	0.64
T6	1.50	0.19	66.99	5.74	131.67	1.60	48.24	5.15
T7	1.50	0.19	71.00	1.54	135.86	6.98	47.27	3.68
T8	1.50	0.30	93.77	2.12	106.29	0.99	38.23	1.31
T9	1.85	0.08	99.63	8.23	114.88	0.37	40.33	0.97
T10	1.85	0.38	90.25	1.83	119.94	1.03	39.06	4.33
T11	2.00	0.19	112.67	2.74	131.04	5.16	41.70	1.10

**Table 5:** Standards equations for elasticity modulus.

Equations		Standard	Concrete Type
$E_{ci}=\alpha_{E}5600\surd f_{ck}$	(2)	NBR 6118, Strength 20 MPa à 50 MPa [38]	Conventional
$E_{ci}=21.5x10³\alpha_{E}{\left(\frac{f_{ck}}{10}+1.25\right)}^{1/3}$	(3)	NBR 6118, Strength 55 MPa à 90 MPa [38]	High Strength
$E_{ci}=4069\surd f_{c}\prime$	(4)	Graybeal (2019) [36]	UHPC
$E_{ci}=4730\surd f_{c}\prime$	(5)	ACI 318 14 [39]	High Strength
$E_{ci}=21.5x10³\alpha_{E}{\left(\frac{f_{ck}+8}{10}\right)}^{1/3}$	(6)	FIB Model Code 2010 [40]	High Strength
$E_{ci}=1.05x22{\left(f_{cm}/10\right)}^{0,3}$	(7)	Eurocode 2 (2010) [41]	High Strength
$E_{ci}=4150\surd f_{c}\prime$	(8)	Sritharan 2003 [37]	UHPC

**Table 6:** Characteristics values of compression strength (MPa).

	Average Strength (*f_cm_*)	Standard Deviation	$f_{ck}$	$f_{c}^{\prime }$
T1	122.94	3.28	117.53	107.38
T2	110.13	3.88	103.72	95.73
T3	124.21	6.56	113.39	108.53
T4	125.54	4.80	117.63	109.74
T5	106.00	1.00	104.35	91.98
T6	131.67	2.22	128.01	115.31
T7	135.86	9.26	120.58	119.12
T8	106.29	1.38	104.01	92.24
T9	114.88	0.50	114.05	100.05
T10	119.94	1.39	117.64	104.65
T11	128.13	7.72	115.39	112.10

**Table 7:** Experimental and estimated values of the elasticity modulus.

	Exp. Average	NBR 6118 (2023) [19] Class I	NBR 6118 (2023) [19] Class II	Graybeal (2019) [17]	ACI 318 (2014) [20]	Model Code (2010) [21]	Eurocode 2 (2010) [22]	Sritharan (2003) [18]
T1	46.14	60.71	50.56	42.16	49.01	49.97	49.04	43.00
T2	41.44	57.03	48.70	39.81	46.28	48.06	47.44	40.60
T3	43.63	59.63	50.01	42.39	49.28	49.41	49.19	43.23
T4	46.08	60.73	50.57	42.63	49.55	49.98	49.35	43.47
T5	40.90	57.20	48.79	39.02	45.36	48.15	46.90	39.80
T6	48.24	63.36	51.88	43.69	50.79	51.32	50.06	44.56
T7	47.27	61.49	50.95	44.41	51.62	50.37	50.53	45.29
T8	38.23	57.11	48.74	39.08	45.43	48.10	46.94	39.86
T9	40.33	59.80	50.10	40.70	47.31	49.50	48.05	41.51
T10	39.06	60.74	50.57	41.63	48.39	49.98	48.67	42.45
T11	41.70	60.16	50.28	43.08	50.08	49.68	49.65	43.94

NB: The $\alpha_{E}$ parameter of NBR 6118:2023 [38] was considered 1.0 because it is closer to experimental values.

**Table 8:** ANOVA analysis for compression strength and elasticity modulus.

Factor	Compression Strength		Elasticity Modulus	
	F	*p*	F	*p*
Steel (L)	0.013524	0.911946	1.465011	0.280225
Steel (Q)	0.010471	0.922472	0.744908	0.427543
PVA (L)	0.219450	0.659171	1.314020	0.303548
PVA (Q)	0.840127	0.401408	1.575318	0.264903

**Table 9:** Standard vs. experimental average of elasticity modulus.

$\frac{E_{exp}}{E_{standard}}$	NBR 6118 (2023) [19] Class I	NBR 6118 (2023) [19] Class II	Graybeal (2019)—UHPC [17]	ACI 318 (2014) [20]	FIB Model Code (2010) [21]	Eurocode 2 (2010) [22]	Sritharan (2003)—UHPC [18]
T1	0.76	0.91	1.09	0.94	0.92	0.94	1.07
T2	0.73	0.85	1.04	0.90	0.86	0.87	1.02
T3	0.73	0.87	1.03	0.89	0.88	0.89	1.01
T4	0.76	0.91	1.08	0.93	0.92	0.93	1.06
T5	0.71	0.84	1.05	0.90	0.85	0.87	1.03
T6	0.76	0.93	1.10	0.95	0.94	0.96	1.08
T7	0.77	0.93	1.06	0.92	0.94	0.94	1.04
T8	0.67	0.78	0.98	0.84	0.79	0.81	0.96
T9	0.67	0.80	0.99	0.85	0.81	0.84	0.97
T10	0.64	0.77	0.94	0.81	0.78	0.80	0.92
T11	0.72	0.86	0.97	0.91	0.87	0.89	0.95
Average	0.72	0.86	1.03	0.89	0.87	0.89	1.01

**Table 10:** Parameters for tensile strength in the elastic phase ($f_{ct,el}$).

Specimen Mean Size (mm)	Yield Point	$f_{ct,fl}$ (MPa)	$f_{ct,el}$
a = 100.215 (width)	F = 40.67 kN	13.96	9.32
b = 100.105 (depth)	Displ. = 0.106166 mm		

## Data Availability

Data is contained within the article.
